# Retraction Note: Recent advances and challenges in single cell protein (SCP) technologies for food and feed production

**DOI:** 10.1038/s41538-026-00718-6

**Published:** 2026-01-20

**Authors:** Yu Pin Li, Fatemeh Ahmadi, Khalil Kariman, Maximilian Lackner

**Affiliations:** 1 College of Agricultural Resources and Environmental Science, Yunnan Agricultural University, Kunming, 650201 China; 2 School of Agriculture and Environment, University of Western Australia, Crawley, WA 6009 Australia; 3 Circe Biotechnologie GmbH, Kerpengasse 125, 1210 Vienna, Austria

**Retraction Note to:** ***npj Science of Food*** 10.1038/s41538-024-00299-2, **published online 18 September 2024**

The Editors-in-Chief have retracted this review article after concerns were raised regarding the validity of several cited references. Upon further investigation, the Publisher identified a mismatch between the article’s text and its cited references, such as 147, 153, 163, 171 and 180. Additionally, Figure 2 referenced analyses that were not present in the article’s content. The authors were unable to provide an explanation for these concerns and submitted a revised reference list, which the Publisher found to contain several discrepancies.

As a result, the Editors-in-Chief no longer have confidence in the reliability of the content of this article.

Fatemeh Ahmadi stated that they and Maximilian Lackner disagree with this retraction. Khalil Kariman and Yu Pin Li disagree with this retraction.

